# Practice patterns for the radical treatment of nasopharyngeal cancer by head and neck oncologists in the United Kingdom

**DOI:** 10.1259/bjr.20170590

**Published:** 2018-02-08

**Authors:** Imran Petkar, Shreerang Bhide, Kate Newbold, Kevin Harrington, Chris Nutting

**Affiliations:** 1 Head and Neck Unit, The Royal Marsden NHS Foundation Trust, London, UK; 2 Radiotherapy and Imaging, The Institute of Cancer Research, Sutton, UK

## Abstract

**Objective:**

Advances in radiation delivery, imaging techniques, and chemotherapy have significantly improved treatment options for non-metastatic nasopharyngeal cancers (NPC). However, their impact on the practice in the United Kingdom (UK), where this tumour is rare, is unknown. This study examined the current attitudes of UK head and neck oncologists to the treatment of NPC.

**Methods:**

UK head and neck oncologists representing 19/23 cancer networks were sent an invitation email with a personalised link to a web-based survey designed to identify the influence of tumour and nodal staging on current NPC management practices.

**Results:**

26/42 (61%) of clinicians responded. Induction chemotherapy followed by concomitant chemoradiation was the treatment of choice for Stage III (69%) and IVa/b (96%), with cisplatin and 5-fluorouracil combination being the most commonly used induction chemotherapy regimen (88%). 16 centres (61%) used a geometric approach, adding variable margins of 0–10 mm to the gross tumour volume to define their therapeutic dose clinical target volume. 54% of respondents used 3 radiotherapy (RT) prescription doses to treat NPC. Retropharyngeal nodal region irradiation policy was inconsistent, with nearly one-quarter treating the entire group to a radical dose.

**Conclusion:**

Significant heterogeneity currently exists in the RT practice of NPC in the UK. A consensus regarding the optimal curative, function-sparing treatment paradigm for NPC is necessary to ensure cancer survivors have satisfactory long-term health-related quality of life.

**Advances in knowledge:**

This is the first study to highlight the significant variation in RT practice of NPC in the UK.

## INTRODUCTION

Primary intensity-modulated radiotherapy (IMRT), either alone or in combination with systemic chemotherapy, forms the mainstay of curative treatment for nasopharyngeal cancers (NPC), achieving 2-year loco-regional control and overall survival rates of 86% in the UK.^1^ The addition of concomitant chemotherapy to radiotherapy (RT) confers an absolute 10 year survival advantage of 9.9% over RT alone in locally advanced Stage III/IVb NPC,^2^ and is the recommended standard of care in the recently published UK multidisciplinary national guidelines.^3^ The role of induction chemotherapy (IC) or adjuvant chemotherapy (AC), in addition to concomitant chemoradiation (CRT), to improve survival outcomes remains contentious, with conflicting results from several meta-analyses.^2,4–8^ Despite such uncertainty, current international guidance supports the use of IC or AC in locally advanced tumours, provided the delivery of definitive RT is not compromised.^9^ At present, it is unclear whether either of these strategies is integrated into routine UK practice for NPC.

Conventional RT target volumes for NPC are large in the UK, in which the entire nasopharyngeal compartment, retropharyngeal lymph nodes (RPN) and involved nodal levels are treated to a radical dose of 65 Gy while uninvolved bilateral Ib–V neck nodes are irradiated to a prophylactic RT dose of 54 Gy, delivered over 30 fractions. However, these therapeutic approaches adversely impact on the long-term functional quality of life in cancer survivors due to treatment-related toxicities, especially xerostomia, dysphagia and damage to the temporal lobe.^10, 11^ NPC is rare in the UK,^3^ and consequently there is a lack of motivation to refine such established curative, but considerably morbid management strategies. On the other hand, a number of currently accruing toxicity-mitigating trials in oro- and hypo-pharyngeal cancers^12–14^ have the potential to shape future organ-sparing protocols in these tumours. Such a notable disparity in the national research portfolio could potentially dichotomise long-term functional outcomes for pharyngeal cancer patients, and there is, therefore, an urgent unmet need to establish a consensus on safe, less morbid RT strategies for NPC in the UK. Potentially favourable evidence-based RT protocols^15^ have been implemented in Asia, where this tumour is endemic. Geographical variations in prognostic epidemiological patterns of NPC,^16^ such as the significant prevalence of Epstein-Barr virus–associated good prognosis non-keratinizing, undifferentiated carcinoma in the Far East compared to the Western World, appear to limit the incorporation of similar treatment options into standard treatment protocols in non-endemic regions such as North America and UK.

This study aims to understand the variation in current NPC management amongst the different UK centres.

## Methods and materials

We performed an observational, web-based survey, which comprised 19 questions exploring local treatment protocols for the management of non-metastatic NPC, with particular emphasis on radiotherapy practice for different tumour stages. It was developed using Bristol Online Survey—a survey tool available on www.onlinesurvays.ac.in. The final version of the survey (Supplementary Material 1, Supplementary material available online) and accompanying protocol were scientifically reviewed and approved as a service evaluation by the Royal Marsden NHS Foundation Trust Clinical Committee for Research (SE531).

The personalised link to the survey was distributed via an invitation email to 42 UK head and neck (HN) oncologists representing 19 of the 23 cancer research networks who had expressed an interest in either ARTDECO (ISRCTN 01483375)^17^ or DARS (ISRCTN 25458988)^12^-two Cancer Research UK (CRUK)-funded multicentre HN randomised controlled trials. In order to maintain anonymity of responding clinicians, no identifiable information was captured in the survey responses. The survey was available for completion for a period of 4 weeks in June and July 2016.

### Statistical analysis

Descriptive statistics were used. Results were collated and presented as percentages to determine the influence of tumour and nodal staging on management practices.

## Results

### Participants

Clinicians from 26 centres (61%) completed the survey. Of those who responded, 92% (*n* = 24/26) completed the questionnaire fully. Centre location, referral numbers and imaging modalities used to stage NPC are included in Table 1.

**Table 1.** Centre location, number of radical treatments of NPC per year, and staging imaging used for NPC

	Number (percentages)
Region	
England	22 (84%)
Wales	2 (8%)
Scotland	1 (4%)
Northern Ireland	1 (4%)
NPC treated radically each year	
0–5	19 (73%)
6–10	7 (27%)
Staging imaging modality	
MRI	2 (8%)
PET-CT	1 (4%)
MRI + PET-CT	4 (16%)
MRI + CT	10 (38%)
MRI + CT + PET-CT	9 (34%)

PET-CT, positron emission tomography; NPC, nasopharyngeal cancers.

### Treatment strategy as per stage

**Figure 1.**
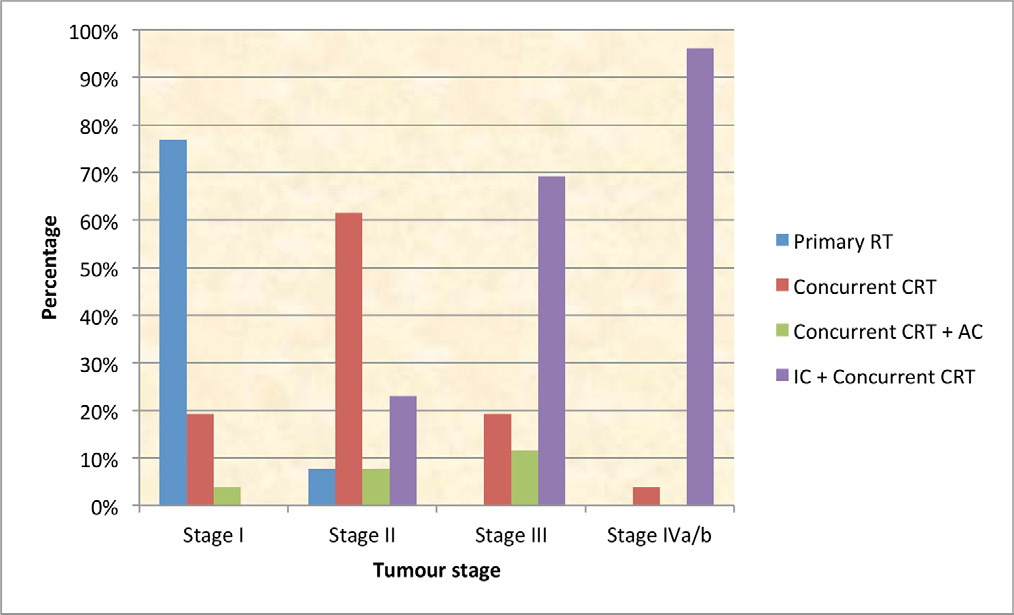
UK centres’ treatment strategy for NPC by stage (*n* = 26).

Disease was staged according to the 2010 American Joint Committee on Cancer (AJCC) criteria. 20/26 (77%) of centres stated that they offered primary RT alone for Stage I NPC (20/26; 77%), while patients with Stage II NPC were more likely to be treated with concurrent CRT (16/26; 61%). IC followed by CRT was the treatment of choice for Stage III (18/26; 69%) and Stage IVa/b NPC (25/26; 96%) (Figure 1).

### Chemotherapy practice

Only one centre does not subscribe to the use of IC or AC, offering primary RT for Stage I NPC and concurrent CRT for Stage II–IVa/b NPC. With regards to IC, 22/25 centres (88%) prescribed cisplatin and 5-fluorouracil (CF), with 10 and 12 of these centres offering 2 and 3 cycles of CF respectively. The remaining 3 centres administered 2 cycles of induction cisplatin, 5-FU and docetaxel (TPF).

Two centres offered 3 cycles of adjuvant CF following concurrent CRT for Stage III NPC, with one of these centres also routinely prescribing AC for Stage II NPC.

### Radiotherapy practice

Rotational arc-based IMRT was widely used (20/26; 77%) to deliver radical RT for NPC, with the remaining centres using fixed-field IMRT. No centre practised conventional 3-D conformal RT. Half of UK centres completing the survey used MRI in radiotherapy planning position co-registered with the planning CT scan to aid delineation of RT target volumes and organs at risk.

### Dose prescription

A large proportion of centres (14/26; 54%) employed 3 dose-volumes: radical dose of 70 Gy (or equivalent), intermediate dose 60 Gy and elective dose of 54–56 Gy for Stage III/IVb NPC. 11 centres (42%) used 2 dose-volumes: radical dose of 65 Gy, and elective dose of 54 Gy, delivered over 30 fractions. One centre stated its standard practice was to use 4 dose-levels—prescribing 70 Gy to the primary tumour, 66 Gy to the remaining NPC and any involved lymph node (LN) regions, 60 Gy and 54 Gy to “high risk” and “low risk” LN respectively; all delivered over 33 fractions.

### Therapeutic dose clinical target volume (CTV) selection

The majority of responding centres (16/26; 61%) defined their therapeutic CTV using a geometric approach, while the remaining 10 centres adopted an anatomical approach and irradiated the entire nasopharynx to a radical RT dose. Amongst those centres that employed a geometric approach, seven added a margin of 10 mm to both primary and nodal gross tumour volumes (GTVs) to create the CTV. Corresponding primary and nodal GTV–CTV margins for the other centres were as follows: 5 mm and 10 mm (4/16), 6 mm (1/16), 5 mm (1/16), 0 mm and 10 mm (1/16), 10 mm and entire nodal level (1/16), and variable margins and entire nodal level (1/16).

### Prophylactic dose nodal-CTV selection

To standardise survey responses, level II was the chosen site of nodal disease that would be irradiated to a radical dose in node-positive NPC.

**Figure 2.**
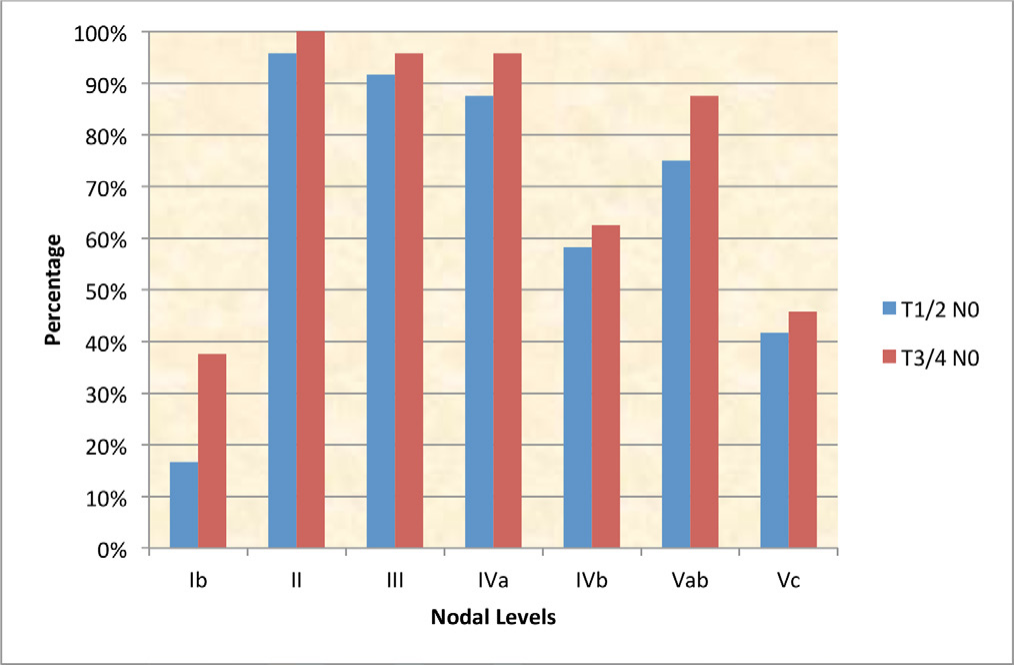
Cumulative prophylactic dose nodal-CTV irradiation policy in node-negative NPC (*n* = 24). CTV, clinical target volume; NPC, nasopharyngeal cancers.

**Figure 3.**
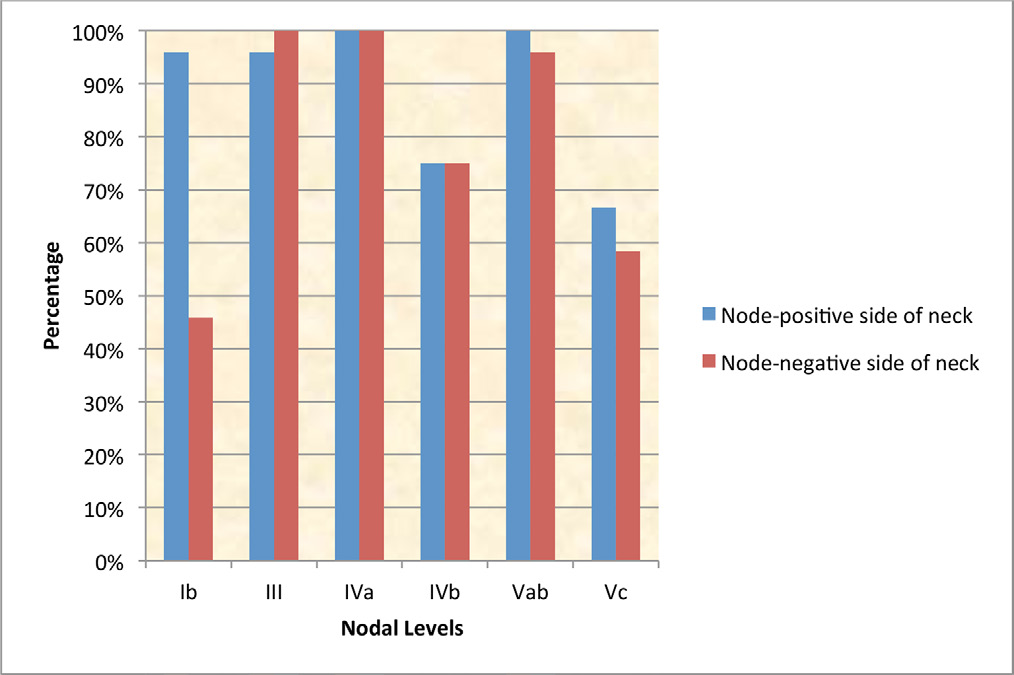
Cumulative prophylactic dose nodal-CTV irradiation policy in node-positive NPC (*n* = 24).

For *T1*/2 node-negative NPC, most centres irradiated level IVa electively (IVa - 21/25, 84%) in addition to levels II, III and Va; however there was uncertainty about including levels IVb (14/25, 56%), and Vc (supraclavicular fossa, SCF; 10/25, 40%) respectively. One centre stipulated that it did not prophylactically irradiate any nodes in this stage, reserving it for T3/4 node-negative tumours only. In comparison, level Ib was treated more frequently in T3/4 node-negative NPC (Figure 2). RT nodal coverage was much more extensive for node-positive disease, with most centres treating the entire neck and nearly all included level Ib on the node-positive side of the neck (Figure 3).

### RPN volume definition and dose prescription

18 centres (69%) included both the medial and lateral groups of RPN in the RT target volumes, and seven centres (23%) treated this group along its entire length (upper edge of body of C1/hard palate to cranial edge of hyoid bone) to a dose equivalent to 70 Gy (radical dose).

## Discussion

The results from this survey illustrate significant variability in the management of NPC in the UK. These include differences in radical RT target volumes, selection of prophylactic dose nodal-CTV and RPN volumes, and the role of systemic chemotherapy in different stages of NPC.

The routine integration of MRI in the RT planning process has improved the accuracy of primary tumour evaluation with its excellent soft tissue discrimination.^18^ This has overcome the widely acknowledged shortcomings associated with CT-based GTV delineation^19–21^ that justified the historical inclusion of the entire nasopharyngeal compartment in the therapeutic radiation dose. A direct consequence of advances in both imaging and RT delivery has been the delineation of smaller therapeutic dose CTV in NPC. Such target volumes, which are generated by adding variable margins to GTV, minimise the risk of long-term treatment-related toxicities without any detrimental impact on survival outcomes. To avoid the risk of geographical misses in the era of highly conformal IMRT, however, we feel that rigid co-registration of MRI in the RT planning position with a similarly acquired planning CT is an essential pre-requisite for reliable tumour delineation.^22^ Resource limitations mean that this facility is not readily available in all UK radiotherapy centres.

**Figure 4.**
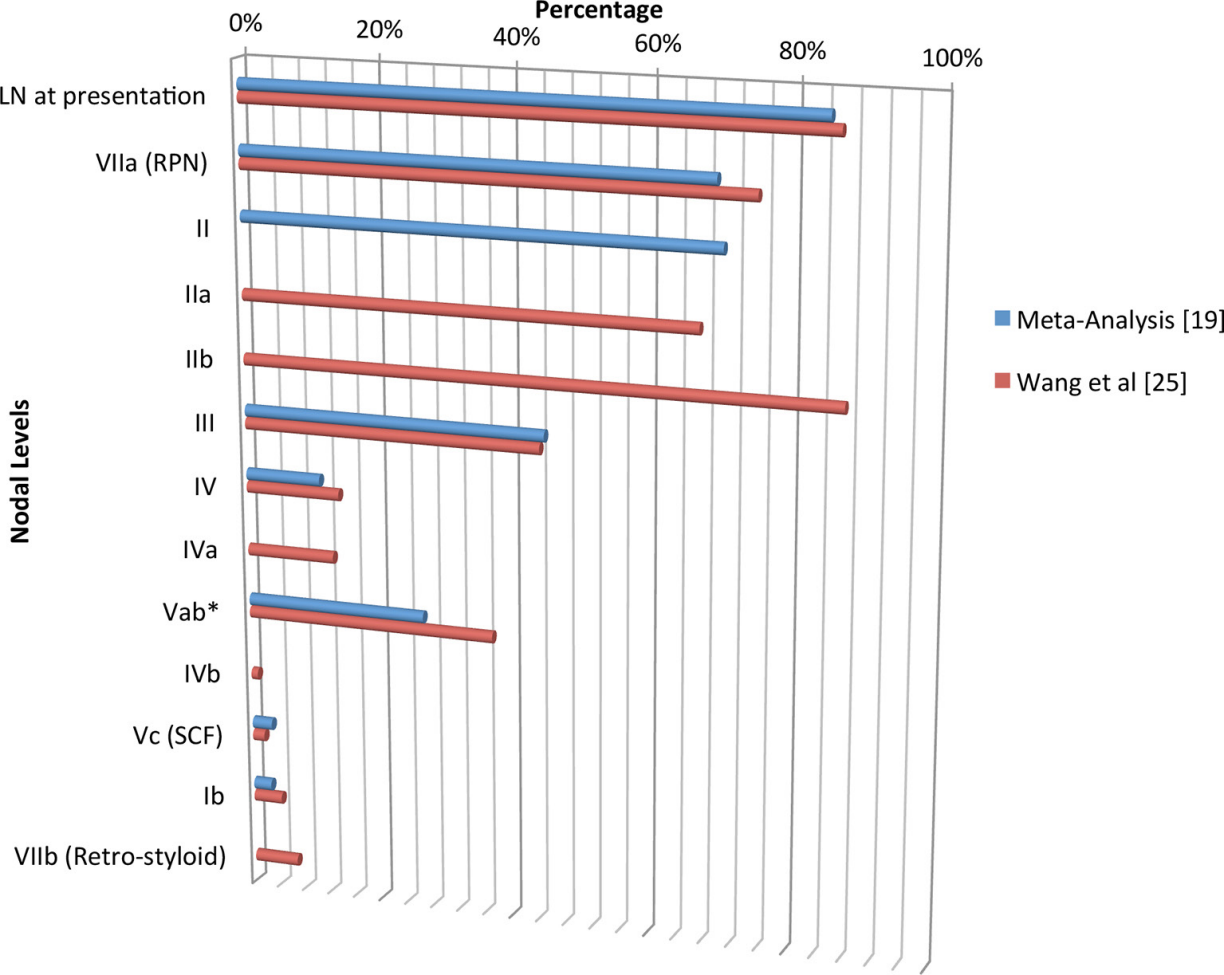
Patterns of regional lymph node metastasis from NPC, *Incidence of nodal metastasis in Va and Vb were 21.5 and 15.6% respectively in Wang et al. NPC, nasopharyngeal cancers.

Strong evidence from MRI-based imaging studies in NPC-endemic regions have refined current understanding of distribution of nodal metastasis (Figure 4), concluding that the disease spread is orderly, with a preferential predilection to spread to the upper cervical nodes (levels II, III, and Va), and that the likelihood of “skip” metastasis is rare.^19,23–25^ Such studies support the introduction of a personalised, selective nodal irradiation strategy, which is likely to be less toxic. Li et al successfully explored this proof of principle in a Phase III, non-inferiority, randomised controlled trial comparing prophylactic upper neck radiation alone against whole neck radiation in node-negative NPC.^26^ Their study, in which nearly two-thirds of patients had T3/4 tumours, demonstrated equivalent 3 year survival [89.5 *vs* 87.4%, hazard ratio (HR) 0.86, 95% confidence Interval (CI) (0.41–1.82), *p* = 0.70] and relapse–free rates [89.8 *vs* 89.3%, HR 0.91, 95% CI (0.42–2.00), *p* = 0.82] in the two arms, thereby confirming that prophylactic irradiation of upper nodal levels was adequate in node-negative non-keratinizing, undifferentiated NPC. Sparing the lower neck, and level Ib is likely to have a favourable influence on patients’ health-related quality of life (HR-QoL), by reducing the risk of long-term thyroid dysfunction, carotid artery atherosclerosis, apical lung fibrosis, skin fibrosis and xerostomia.

RPN metastasis in NPC is common, occurring in nearly three-quarters of newly diagnosed cases. It is also associated with worsened survival outcomes,^27^ leading to empirical irradiation of this nodal group in NPC, regardless of stage at presentation. Evidence-based delineation of this LN group, along with selecting the most appropriate RT dose are crucial, however, in order to reduce long-term treatment-related morbidity. In this regard, it is noteworthy that involvement of the medial group of RPN is extremely rare (<1%) in NPC, and always associated with other sites of nodal metastasis, as highlighted by a large prospective MRI-based study.^28^ Further corroboration is delivered by the recent publication of the updated international consensus nodal delineation guidelines, in which the medial RPN is excluded from level VIIa.^29^ Sparing the medial RPN reduces the RT dose to the pharyngeal constrictor muscles, a key swallowing structure implicated in post-RT dysphagia, and may minimise long-term swallowing dysfunction.^30^ Furthermore, successive studies have failed to demonstrate significant differences in outcomes between tumours involving RPN and those with neck nodes < 6 cm above SCF—both of which are staged as N1 in the current NPC staging system.^31, 32^ The rationale, therefore, to irradiate RPN to a radical dose while including levels II–III in the elective dose, when these nodal regions are free of macroscopic disease, is flawed.

The rationale for adding IC to CRT in NPC is based mainly on the premise that it reduces rates of distant metastasis through early elimination of micro-metastatic disease, tumour shrinkage can occur before RT, and treatment compliance is better compared to AC. Such anticipated benefits, however, have failed to translate into clear survival advantages over CRT alone in the published meta-analyses, and consequently IC is not recognised globally as the therapeutic option of choice for Stage III–IVb NPC. Nonetheless, a recently reported comprehensive individual patient data network meta-analysis (NMA) by Blanchard et al showed that CRT was non-significantly inferior to IC followed by CRT for PFS, loco-regional and distant control,^33^ which suggests that there may be a beneficial role for IC in carefully selected locally advanced NPC. NPC patients in the UK are more likely to present with a primary tumour volume >25 cm,^31^ which has been linked with worsened survival and distant recurrence rates.^34^ Therefore, it is plausible to postulate that patients with predominantly bulky disease in the UK benefit from IC, as demonstrated in our Phase II study.^1^ Furthermore, a recently published Phase III randomised study by Sun et al reported a significantly improved 3 year failure-free survival [80 *vs* 72%; HR 0.68, 95 % CI (0.48–0.97); *p* = 0.034], overall survival [92 *vs* 86%; HR 0.59, 95% CI (0.36–0.95); *p* = 0.029], and distant failure-free survival [90 *vs* 83%; HR 0.59, 95% CI (0.37–0.96); *p* = 0.031] rates when dose-reduced induction TPF was added to CRT, compared to concomitant CRT alone.^35^ With regards to AC, again there are no proven survival advantages over CRT in the published literature. The NMA by Blanchard et al did show that it achieved the highest survival benefit amongst the different regimens for NPC and is, therefore, an acceptable regimen.

How much evidence-based is radical NPC treatment in the UK? Nearly 40% (10/26) of respondents in this survey irradiated the entire compartment to a radical dose, though half of these centres (5/10) did not have a planning MRI at their disposal, which is likely to be a contributory factor for not reducing target volumes. The remaining five centres routinely had a planning MRI at their disposal; yet they selected an anatomical approach to define their radical dose. We would consider their approach as very conservative, and not in keeping with international practice, where the GTV is expanded by upto 5 mm to generate the high-dose CTV.^36–38^ Interestingly, 8 of the 16 centres that employed a geometric approach to select their therapeutic CTV did not utilise a RT planning MRI. Instead, they relied on the diagnostic MRI for the purpose of tumour delineation. This methodology is associated with considerable inter- and intra-observer variation, and in our opinion not reliable for accurate target volume delineation, though there may be a role for using deformable registration to integrate the diagnostic MRI and minimise contouring errors in such situations.^39^

This survey additionally demonstrated that prophylactic nodal (including RPN) CTV selection in the UK was extensive, inconsistent and not evidence-based in node-negative NPC, with many centres reluctant to exclude the lower neck, while some erroneously believed that a higher T-stage was associated with a higher risk of microscopic disease in levels Ib and the lower neck (Table 2). Volume selection amongst responding centres in node-positive disease was largely non-selective (Table 3), reflecting traditional UK perception that NPC is a tumour with an aggressive propensity for loco-regional spread and, therefore, justifying the negative culture of a blanket nodal RT target volume policy.^3^ Medial RPN is frequently included in target volumes, with almost a quarter irradiating the entire RPN to a radical dose. Careful re-evaluation of such assumptions are necessary, particularly in an era where there is an increasing drive to develop a personalised, toxicity-sparing approach to manage tumours. One could argue that the reported positive outcomes from NPC-endemic regions, where good-prognosis, virus-associated non-keratinising, undifferentiated (Type III) NPC predominates, cannot be readily extrapolated to the non-viral, keratinising (Type I) and non-keratinising well differentiated (Type II) NPC commonly diagnosed in the Western world.^16^ However, this distinction in the global distribution of prognostic NPC histological sub-types is not absolute. Recent literature suggests that Type III NPC constituted between 55–65% of newly diagnosed NPC in the UK, and upto 30% of such patients might have no nodal disease at presentation.^1, 40^ We feel that a more pragmatic approach, therefore, would be to adopt the favourable selective nodal irradiation policy in Type III, node-negative tumours, while reserving the standard protocol for node-positive disease. In fact, evidence is also gradually emerging regarding the feasibility of irradiating reduced nodal volumes in node-positive NPC,^41, 42^ but this approach still requires prospective validation prior to adopting into clinical practice.

**Table 2.**
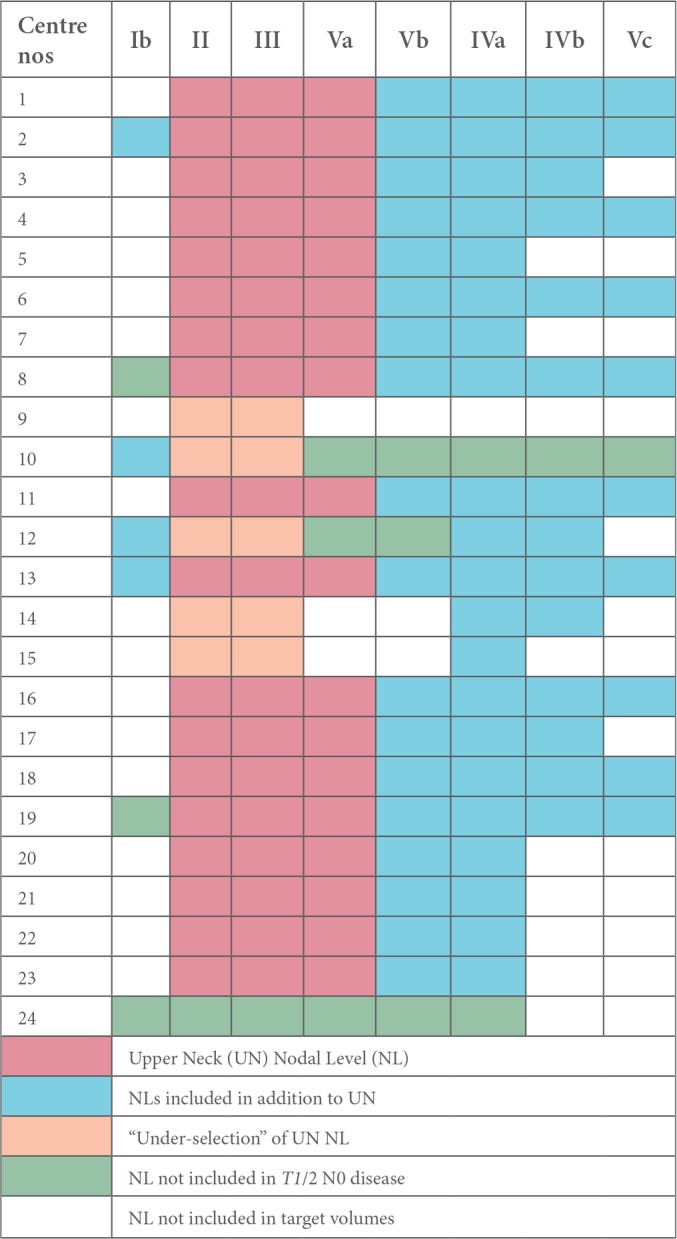
UK centres’ prophylactic dose nodal-CTV selection for node-negative NPC

CTV, clinical target volume; NPC, nasopharyngeal cancers.

**Table 3.**
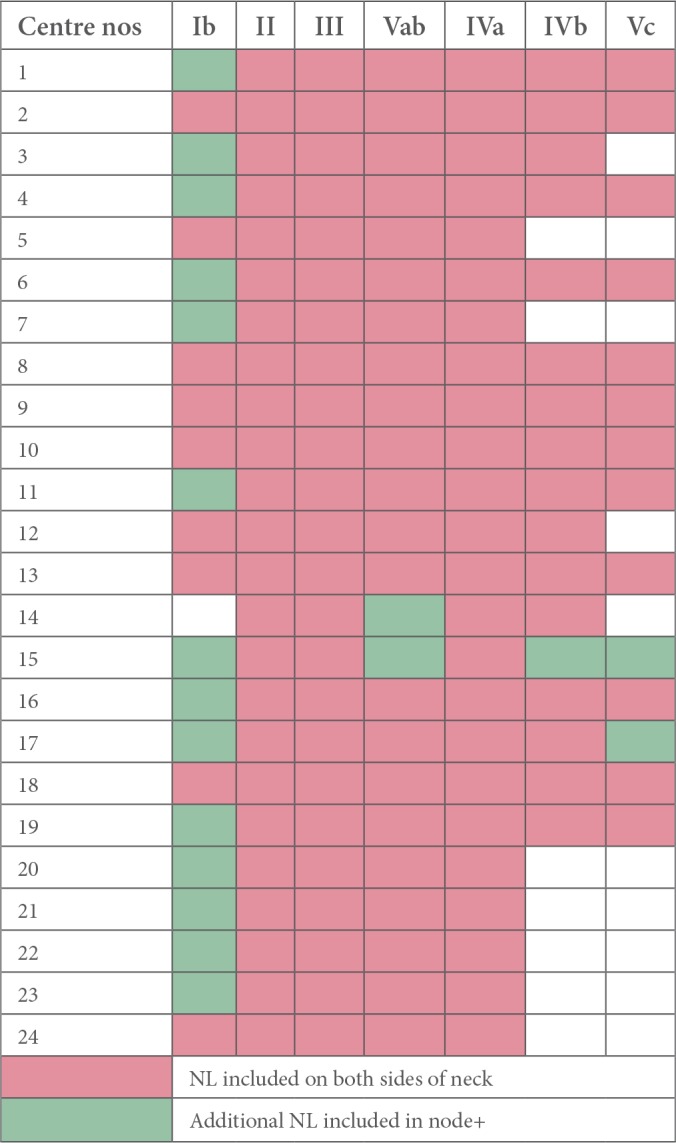
UK centres’ prophylactic dose nodal-CTV selection for node-positive NPC

CTV, clinical target volume; NPC, nasopharyngeal cancers.

The use of IC in the radical treatment of NPC is a popular strategy in the UK. Long-term toxicity and survival outcomes from the study by Sun et al, together with results from additional IC trials^16^ should clarify, in the future, the definitive role of IC in the management of NPC. Until these results are available, it is likely that IC will continue to form a significant part of the treatment strategy in the UK for locally advanced NPC. In this context, we would like to point out that the 2 IC regimens used in the published positive IC trials in NPC were 3 and 2 cycles of TPF and cisplatin-docetaxel, respectively. Currently, the evidence is lacking regarding the use of 2–3 cycles of induction PF in NPC, as prescribed by a majority of responding UK centres. The issue of whether to use pre- or post- IC tumour volumes for RT delineation was investigated in a randomised study which showed that the use of post-IC volumes were associated with a significant improvement in HR-QoL with no detrimental impact on survival outcome.^43^ The use of AC is rare in the UK, reflecting concerns about toxicity and compliance with this approach.

There are certain limitations to this survey. The response rate was 61% and it is probable that non-responding centres may have a different opinion on the management strategy, resulting in selection bias. The response rate is, however, comparable to similar surveys undertaken for other HNC and tumour sites in the UK.^44, 45^ Furthermore, only the local principal investigator (PI) from each centre taking part in either ARTDECO or DARS was invited to take part in the survey, and it can be argued that their treatment paradigm may not necessarily be representative of other HN oncologists at their respective centre. However, we believe it is routine practice for UK cancer centres to have local chemotherapy and RT guidelines for various tumour sites, and there is a high likelihood that oncologists in one centre will uniformly adhere to those guidelines. Therefore, it is reasonable to assume that the practice policy of each PI is reflective of other HN oncologists at their centre. RT contouring variation between HN oncologists in a centre may occur, but significant differences are more likely with complex clinical cases.^46^ This was not the aim of this survey, which was undertaken to understand the variability in standard practice patterns for different stages of non-metastatic NPC. We also did not explicitly explore the influence of the T-stage on management practice for node-positive NPC. Finally, the survey did not specifically enquire whether centres stratified their practice as per the histological sub-type. However, none of the responding centres highlighted this in the several free-text opportunities provided within the survey, and it can, therefore, be assumed with a degree of certainty that centre policy was independent of histology.

## Conclusions

The contemporary management for non-metastatic NPC represents a significant clinical enigma, requiring a balance between curative and function-preserving strategies. This survey demonstrates that significant challenges exist in the UK to achieve this, as highlighted by the large variation in target volume selection amongst different centres. Optimal application of available imaging techniques to better define CTV, selective nodal irradiation in node-negative tumours, prospective peer review of target volumes,^47^ and evidence-based practice for the use of chemotherapy across all NPC stages should be considered to improve current practice (Table 4). Developing a RT consensus for NPC in the UK is essential, and in this regard, we welcome the recent endeavour of our colleagues to initiate this,^48^ and expect that this survey will provide definitive information to finalise a national consensus.

**Table 4.** International guidelines for target volume selection in NPC

	RTOG 0225^38^	DAHANCA^36^	NPC-endemic regions^37, 43^
CTV1 (high dose)	GTV + 0 mm	GTV + 5 mm	GTV + 0 mm
CTV2 (intermediate dose)	CTV1 + 5 mm; to include NPX, soft palate, clivus, skull base, PF, PPS, inferior sphenoid sinus, posterior third of nasal cavity and maxillary sinus, elective nodal levels	CTV1 + 5 mm; to include NPX, skull base, choana	CTV1 + 0.5–1 cm and 5 mm submucosal volume; to include NPX
CTV3 (low dose)	Low neck (levels IV, Vb) may be included in node-negative neck in CTV3 rather than CTV2	Elective nodal levels	CTV2, posterior nasal cavity, posterior maxillary sinus, PF, PPS, skull base, clivus, elective nodal levels
Node-negative NPC	Bilateral II–V, VIIa in CTV2	Bilateral II–V, VIIab in CTV3	II–III, Va, VIIa
Node-positive NPC	Bilateral 1b–V, VIIa in CTV2	Bilateral II–V, VIIab in CTV3	II–V, VIIa

NPX, nasopharynx; PF, pterygopalatine fossa; PPS, Parapharyngeal space.

CTV, clinical target volume; NPC, nasopharyngeal cancers.

## References

[1] Miah AB, Bhide SA, Del Rosario L, Matthews J, Nicol R, Tanay MA, et al Induction chemotherapy followed by chemo-intensity-modulated radiotherapy for locally advanced nasopharyngeal cancer. Clin Oncol 2016; 28: e61–e67. doi: 10.1016/j.clon.2016.01.012 26876458

[2] Blanchard P, Lee A, Marguet S, Leclercq J, Ng WT, Ma J, et al Chemotherapy and radiotherapy in nasopharyngeal carcinoma: an update of the MAC-NPC meta-analysis. Lancet Oncol 2015; 16: 645–55. doi: 10.1016/S1470-2045(15)70126-9 25957714

[3] Simo R, Robinson M, Lei M, Sibtain A, Hickey S Nasopharyngeal carcinoma: United Kingdom national multidisciplinary guidelines. J Laryngol Otol 2016; 130(S2): S97–S103. doi: 10.1017/S0022215116000517 27841121 PMC4873914

[4] Chen YP, Wang ZX, Chen L, Liu X, Tang LL, Mao YP, et al A Bayesian network meta-analysis comparing concurrent chemoradiotherapy followed by adjuvant chemotherapy, concurrent chemoradiotherapy alone and radiotherapy alone in patients with locoregionally advanced nasopharyngeal carcinoma. Ann Oncol 2015; 26: 205–11. doi: 10.1093/annonc/mdu507 25355717

[5] Wang M, Tian H, Li G, Ge T, Liu Y, Cui J, et al Significant benefits of adding neoadjuvant chemotherapy before concurrent chemoradiotherapy for locoregionally advanced nasopharyngeal carcinoma: a meta-analysis of randomized controlled trials. Oncotarget 2016; 7: 48375–90. doi: https://doi.org/10.18632/oncotarget.10237 27356743 10.18632/oncotarget.10237 PMC5217024

[6] Yu H, Gu D, He X, Gao X, Bian X The role of induction and adjuvant chemotherapy in combination with concurrent chemoradiotherapy for nasopharyngeal cancer: a Bayesian network meta-analysis of published randomized controlled trials. Onco Targets Ther 2016; 9: 159–70. doi: 10.2147/OTT.S96983 26793000 PMC4708240

[7] Yan M, Kumachev A, Siu LL, Chan KK Chemoradiotherapy regimens for locoregionally advanced nasopharyngeal carcinoma: a Bayesian network meta-analysis. Eur J Cancer 2015; 51: 1570–9. doi: 10.1016/j.ejca.2015.04.027 26044925

[8] Chen YP, Guo R, Liu N, Liu X, Mao YP, Tang LL, et al Efficacy of the additional neoadjuvant chemotherapy to concurrent chemoradiotherapy for patients with locoregionally advanced nasopharyngeal carcinoma: a Bayesian network meta-analysis of randomized controlled trials. J Cancer 2015; 6: 883–92. doi: 10.7150/jca.11814 26284140 PMC4532986

[9] Chan AT, Grégoire V, Lefebvre JL, Licitra L, Hui EP, Leung SF, et al Nasopharyngeal cancer: EHNS-ESMO-ESTRO Clinical Practice Guidelines for diagnosis, treatment and follow-up. Ann Oncol 2012; 23(Suppl 7): vii83–vii85. doi: 10.1093/annonc/mds266 22997460

[10] Powell C, Schick U, Morden JP, Gulliford SL, Miah AB, Bhide S, et al Fatigue during chemoradiotherapy for nasopharyngeal cancer and its relationship to radiation dose distribution in the brain. Radiother Oncol 2014; 110: 416–21. doi: 10.1016/j.radonc.2013.06.042 23953411

[11] Zhang B, Mo Z, Du W, Wang Y, Liu L, Wei Y Intensity-modulated radiation therapy versus 2D-RT or 3D-CRT for the treatment of nasopharyngeal carcinoma: a systematic review and meta-analysis. Oral Oncol 2015; 51: 1041–6. doi: 10.1016/j.oraloncology.2015.08.005 26296274

[12] Petkar I, Rooney K, Roe JW, Patterson JM, Bernstein D, Tyler JM, et al DARS: a phase III randomised multicentre study of dysphagia- optimised intensity- modulated radiotherapy (Do-IMRT) versus standard intensity- modulated radiotherapy (S-IMRT) in head and neck cancer. BMC Cancer 2016; 16: 770: 770. doi: 10.1186/s12885-016-2813-0 27716125 PMC5052945

[13] Owadally W, Hurt C, Timmins H, Parsons E, Townsend S, Patterson J, et al PATHOS: a phase II/III trial of risk-stratified, reduced intensity adjuvant treatment in patients undergoing transoral surgery for Human papillomavirus (HPV) positive oropharyngeal cancer. BMC Cancer 2015; 15: 602: 602. doi: 10.1186/s12885-015-1598-x 26311526 PMC4549836

[14] Kelly JR, Husain ZA, Burtness B Treatment de-intensification strategies for head and neck cancer. Eur J Cancer 2016; 68: 125–33. doi: 10.1016/j.ejca.2016.09.006 27755996 PMC5734050

[15] Lee AW, Ma BB, Ng WT, Chan AT Management of nasopharyngeal carcinoma: current practice and future perspective. J Clin Oncol 2015; 33: 3356–64. doi: 10.1200/JCO.2015.60.9347 26351355

[16] Chua MLK, Wee JTS, Hui EP, Chan ATC Nasopharyngeal carcinoma. Lancet 2016; 387: 1012–24. doi: 10.1016/S0140-6736(15)00055-0 26321262

[17] Nutting C ART DECO trial management group. First results of ART DECO (CRUK/10/018): a randomised trial of dose escalated intensity modulated radiotherapy (DE-IMRT) versus standard dose IMRT (ST-IMRT) in locally advanced head and neck cancer : NCRI Cancer Conferences Abstracts. Liverpool, UK: The British Institute of Radiology.; 2016.

[18] Chen WS, Li JJ, Hong L, Xing ZB, Wang F, Li CQ Comparison of MRI, CT and 18F-FDG PET/CT in the diagnosis of local and metastatic of nasopharyngeal carcinomas: an updated meta analysis of clinical studies. Am J Transl Res 2016; 8: 4532–47. 27904660 PMC5126302

[19] Ho FC, Tham IW, Earnest A, Lee KM, Lu JJ Patterns of regional lymph node metastasis of nasopharyngeal carcinoma: a meta-analysis of clinical evidence. BMC Cancer 2012; 12: 98. doi: 10.1186/1471-2407-12-98 22433671 PMC3353248

[20] Bird D, Scarsbrook AF, Sykes J, Ramasamy S, Subesinghe M, Carey B, et al Multimodality imaging with CT, MR and FDG-PET for radiotherapy target volume delineation in oropharyngeal squamous cell carcinoma. BMC Cancer 2015; 15: 844. doi: 10.1186/s12885-015-1867-8 26530182 PMC4632362

[21] Njeh CF Tumor delineation: the weakest link in the search for accuracy in radiotherapy. J Med Phys 2008; 33: 136–40. doi: 10.4103/0971-6203.44472 19893706 PMC2772050

[22] Brunt JN Computed tomography-magnetic resonance image registration in radiotherapy treatment planning. Clin Oncol 2010; 22: 688–97. doi: 10.1016/j.clon.2010.06.016 20674300

[23] Castelijns JA, van den Brekel MW Imaging of lymphadenopathy in the neck. Eur Radiol 2002; 12: 727–38. doi: 10.1007/s003300101102 11960218

[24] Ng WT, Lee AW, Kan WK, Chan J, Pang ES, Yau TK, et al N-staging by magnetic resonance imaging for patients with nasopharyngeal carcinoma: pattern of nodal involvement by radiological levels. Radiother Oncol 2007; 82: 70–5. doi: 10.1016/j.radonc.2006.11.010 17166610

[25] Wang X, Hu C, Ying H, He X, Zhu G, Kong L, et al Patterns of lymph node metastasis from nasopharyngeal carcinoma based on the 2013 updated consensus guidelines for neck node levels. Radiother Oncol 2015; 115: 41–5. doi: 10.1016/j.radonc.2015.02.017 25748143

[26] Li JG, Yuan X, Zhang LL, Tang YQ, Liu L, Chen XD, et al A randomized clinical trial comparing prophylactic upper versus whole-neck irradiation in the treatment of patients with node-negative nasopharyngeal carcinoma. Cancer 2013; 119: 3170–6. doi: 10.1002/cncr.28201 23765713

[27] McLaughlin MP, Mendenhall WM, Mancuso AA, Parsons JT, McCarty PJ, Cassisi NJ, et al Retropharyngeal adenopathy as a predictor of outcome in squamous cell carcinoma of the head and neck. Head Neck 1995; 17: 190–8. doi: 10.1002/hed.2880170304 7782203

[28] Wang XS, Yan C, Hu CS, Ying HM, He XY, Zhou ZR, et al Study of the medial group retropharyngeal node metastasis from nasopharyngeal carcinoma based on 3100 newly diagnosed cases. Oral Oncol 2014; 50: 1109–13. doi: 10.1016/j.oraloncology.2014.08.007 25200523

[29] Grégoire V, Ang K, Budach W, Grau C, Hamoir M, Langendijk JA, et al Delineation of the neck node levels for head and neck tumors: a 2013 update. DAHANCA, EORTC, HKNPCSG, NCIC CTG, NCRI, RTOG, TROG consensus guidelines. Radiother Oncol 2014; 110: 172–81. doi: 10.1016/j.radonc.2013.10.010 24183870

[30] Feng FY, Kim HM, Lyden TH, Haxer MJ, Worden FP, Feng M, et al Intensity-modulated chemoradiotherapy aiming to reduce dysphagia in patients with oropharyngeal cancer: clinical and functional results. J Clin Oncol 2010; 28: 2732–8. doi: 10.1200/JCO.2009.24.6199 20421546 PMC2881852

[31] Tang L, Li L, Mao Y, Liu L, Liang S, Chen Y, et al Retropharyngeal lymph node metastasis in nasopharyngeal carcinoma detected by magnetic resonance imaging : prognostic value and staging categories. Cancer 2008; 113: 347–54. doi: 10.1002/cncr.23555 18459174

[32] Tang LL, Guo R, Zhou G, Sun Y, Liu LZ, Lin AH, et al Prognostic value and staging classification of retropharyngeal lymph node metastasis in nasopharyngeal carcinoma patients treated with intensity-modulated radiotherapy. PLoS One 2014; 9: e108375. doi: 10.1371/journal.pone.0108375 25302611 PMC4193733

[33] Ribassin-Majed L, Marguet S, Lee AWM, Ng WT, Ma J, Chan ATC, et al What Is the best treatment of locally advanced nasopharyngeal carcinoma? An individual patient data network meta-analysis. J Clin Oncol 2017; 35: 498–505. doi: 10.1200/JCO.2016.67.4119 27918720 PMC5791836

[34] Wu Z, Su Y, Zeng RF, Gu MF, Huang SM Prognostic value of tumor volume for patients with nasopharyngeal carcinoma treated with concurrent chemotherapy and intensity-modulated radiotherapy. J Cancer Res Clin Oncol 2014; 140: 69–76. doi: 10.1007/s00432-013-1542-x 24173695 PMC11823901

[35] Sun Y, Li WF, Chen NY, Zhang N, Hu GQ, Xie FY, et al Induction chemotherapy plus concurrent chemoradiotherapy versus concurrent chemoradiotherapy alone in locoregionally advanced nasopharyngeal carcinoma: a phase 3, multicentre, randomised controlled trial. Lancet Oncol 2016; 17: 1509–20. doi: 10.1016/S1470-2045(16)30410-7 27686945

[36] Hansen CR, Johansen J, Samsøe E, Andersen E, Petersen JBB, Jensen K, et al Consequences of introducing geometric GTV to CTV margin expansion in DAHANCA contouring guidelines for head and neck radiotherapy. Radiother Oncol 2017; 126: 43–47. doi: 10.1016/j.radonc.2017.09.019 28987748

[37] Lin S, Pan J, Han L, Guo Q, Hu C, Zong J, et al Update report of nasopharyngeal carcinoma treated with reduced-volume intensity-modulated radiation therapy and hypothesis of the optimal margin. Radiother Oncol 2014; 110: 385–9. doi: 10.1016/j.radonc.2014.01.011 24560755

[38] Chen A, Lee N, Yang C, Liu T, Narayan S, Vijayakumar S, et al Comparison of intensity-modulated radiotherapy using helical tomotherapy and segmental multileaf collimator-based techniques for nasopharyngeal carcinoma: dosimetric analysis incorporating quality assurance guidelines from RTOG 0225. Technol Cancer Res Treat 2010; 9: 291–8. doi: 10.1177/153303461000900308 20441239

[39] Chuter R, Prestwich R, Bird D, Scarsbrook A, Sykes J, Wilson D, et al The use of deformable image registration to integrate diagnostic MRI into the radiotherapy planning pathway for head and neck cancer. Radiother Oncol 2017; 122: 229–35. doi: 10.1016/j.radonc.2016.07.016 27497803

[40] Boon CS, Hartley A, Sanghera P Initial efficacy of hypofractionated accelerated chemo-tomotherapy® for nasopharyngeal carcinoma. Clin Oncol 2015; 27: 484–5. doi: 10.1016/j.clon.2015.04.001 25963855

[41] Hu W, Zhu G, Guan X, Wang X, Hu C The feasibility of omitting irradiation to the contralateral lower neck in stage N1 nasopharyngeal carcinoma patients. Radiat Oncol 2013; 8: 230. doi: 10.1186/1748-717X-8-230 24094078 PMC3854105

[42] Seol KH, Lee JE Patterns of failure after the reduced volume approach for elective nodal irradiation in nasopharyngeal carcinoma. Radiat Oncol J 2016; 34: 10–17. doi: 10.3857/roj.2016.34.1.10 27104162 PMC4831964

[43] Yang H, Chen X, Lin S, Rong J, Yang M, Wen Q, et al Treatment outcomes after reduction of the target volume of intensity-modulated radiotherapy following induction chemotherapy in patients with locoregionally advanced nasopharyngeal carcinoma: a prospective, multi-center, randomized clinical trial. Radiother Oncol 2017; 126: 37–42. doi: 10.1016/j.radonc.2017.07.020 28864073

[44] Gujral DM, Piercy D, Morden JP, Emson M, Hall E, Miah AB, et al Current attitudes of head and neck oncologists in the United Kingdom to induction chemotherapy for locally advanced head and neck cancer: a survey of centres participating in a national randomised controlled trial. Oral Oncol 2014; 50: 141–6. doi: 10.1016/j.oraloncology.2013.10.012 24263110

[45] Davda R, Hughes S, Jones R, Crabb SJ, Troup J, Payne H Chemotherapy at first diagnosis of advanced prostate cancer – revolution or evolution? Findings from a British uro-oncology group UK survey to evaluate oncologists' views on first-line docetaxel in combination with Androgen deprivation therapy in castrate-sensitive metastatic and high-risk/locally advanced prostate cancer. Clin Oncol 2016; 28: 376–85. doi: 10.1016/j.clon.2016.01.006 26874654

[46] Fong C, Sanghera P, Good J, Nightingale P, Hartley A Implementing head and neck contouring peer review without pathway delay: the on-demand approach. Clin Oncol 2017; 29: 841–7. doi: 10.1016/j.clon.2017.09.005 28988705

[47] Royal College of Radiologists. Radiotherapy target volume definition and peer review – RCR guidance. 2017 Available from: https://www.rcr.ac.uk/publication/radiotherapy-target-volume-definition-and-peer-review [10/01/2018] 10.1016/j.clon.2019.07.021 31444023

[48] Fong CTK, Boon CS, Tiffany M, Roques T, Brammer C, Foran B, et al UK contouring variation in nasopharyngeal carcinoma. Int J Radiat Oncol Biol Phys 2016; 96(Suppl 2): E393. doi: 10.1016/j.ijrobp.2016.06.1620

